# Nanomaterial-Modified Antibacterial Membranes for Water Treatment: From Dimensional Classification and Modification Strategies to Antimicrobial Mechanisms

**DOI:** 10.3390/membranes16090282

**Published:** 2026-08-24

**Authors:** Lu Pei, Bingrong Wang, Yutong Zheng, Yang Liu, Yang Zhou, Xiangdong Zeng

**Affiliations:** College of Materials and Chemistry & Chemical Engineering, Chengdu University of Technology, Chengdu 610059, China; peilu@stu.cdut.edu.cn (L.P.); wangbingrong@stu.cdut.edu.cn (B.W.); 202402070230@stu.cdut.edu.cn (Y.Z.); liuyang13@stu.cdut.edu.cn (Y.L.); 202402070130@stu.cdut.edu.cn (Y.Z.)

**Keywords:** nanomaterials, antibacterial membranes, biofouling, membrane modification, review and prospects

## Abstract

Membrane separation technology is extensively used in water treatment. However, during long-term operation, biofouling caused by bacteria and other microorganisms significantly limits the service life of membranes. Introducing antibacterial nanomaterials onto the membrane surface or into the internal structure is an effective way to combat biofouling. This review systematically summarizes recent progress in antibacterial membranes modified with different nanomaterials. First, we classify antibacterial nanomaterials by dimensionality and highlight their physicochemical properties and effects on overall membrane performance. Furthermore, we summarize the advantages, disadvantages, and applicability of three antibacterial nanomaterial modification strategies for membranes, including surface coating, grafting, and blending. Subsequently, we analyze in depth the main antibacterial mechanisms that enhance membrane performance, including metal ion release, reactive oxygen species oxidation, physical contact disruption, and anti-adhesion, as well as their synergistic effects. Finally, we critically evaluate the remaining challenges, such as interfacial compatibility between nanomaterials and polymers, controlled release of metal ions, and environmental safety. This review provides a reference for the rational design of high-performance antibacterial membranes.

## 1. Introduction

With the growing shortage of water resources and the increasing severity of water pollution [1,2,3], membrane separation technology has been widely applied in water treatment due to its high efficiency, low energy consumption, and compact footprint [4,5,6]. However, membrane fouling, especially biofouling, remains a major obstacle to its practical application [3,7,8]. Under the influence of gravitational sedimentation and other factors, suspended microorganisms reversibly adhere to the surface of the conditioning layer. The extracellular polymeric substances (EPS) secreted by the microorganisms convert reversible adhesion into irreversible attachment and form microcolonies. Through continuous accumulation and EPS secretion, these microcolonies develop into biofouling films [9,10,11]. During this process, membrane pore blockage causes a gradual increase in transmembrane pressure and a corresponding decrease in permeate flux. In addition, the dense biofilm not only alters the membrane surface charge and hydrophobicity, leading to fluctuations in salt and contaminant rejection rates, but also forces the membrane system to increase operating pressure and shorten chemical cleaning cycles, thus significantly raising operational energy consumption and maintenance costs [12,13,14]. When bactericides cannot penetrate the dense EPS matrix sufficiently, chemical cleaning may be ineffective. Similarly, cell lysis caused by oxidation or chemical cleaning may release intracellular components, including endotoxins from Gram-negative bacteria. This has a significant impact on the quality of treated water.

Conventional surface hydrophilic modification is no longer sufficient to address biofouling [15,16,17]. Therefore, introducing nanomaterials that possess both physical size effects and chemical bactericidal activity has become a key strategy [18,19,20]. With the extremely high specific surface area and surface activity, nanomaterials can endow membranes with significant antibacterial properties at very low loadings [21,22,23], which has made the development of antibacterial membranes a research hotspot in recent years [24,25,26]. They offer diverse antibacterial pathways, ranging from ion release to physical disruption, and chemical oxidation to surface repulsion [3,27,28]. Their dimensions are much smaller than membrane pores or polymer chain segments, allowing them to be incorporated via surface coating, grafting, or blending without destroying the separation layer [29,30,31]. Through the introduction of nanomaterials, the membrane surface transforms from a simple separation interface into a composite barrier with both bactericidal and anti-adhesive functions [32,33,34].

This review summarizes the antibacterial mechanisms of nanomaterial-modified membranes, classifies the nanomaterials by dimension and their applications, outlines the three main incorporation methods, highlights the multiple antibacterial mechanisms and target bacterial species, and discusses current challenges and future prospects.

## 2. Dimensional Classification and Characteristics of Nanomaterials

Nanomaterials are defined as materials with at least one external dimension on the nanoscale, or having internal or surface structures at the nanoscale [29,35]. Examining antibacterial nanomaterials used for membrane modification from a dimensional perspective helps to understand the structure–property relationships and guide the optimization of modification strategies [12,29,31]. According to dimension, these materials can be divided into zero-dimensional (0D), one-dimensional (1D), two-dimensional (2D), and three-dimensional (3D) nanomaterials [34,36,37].

### 2.1. Zero-Dimensional Nanomaterials

#### 2.1.1. Metal Nanoparticles

**Silver Nanoparticles (AgNPs)**

**Figure 1:**
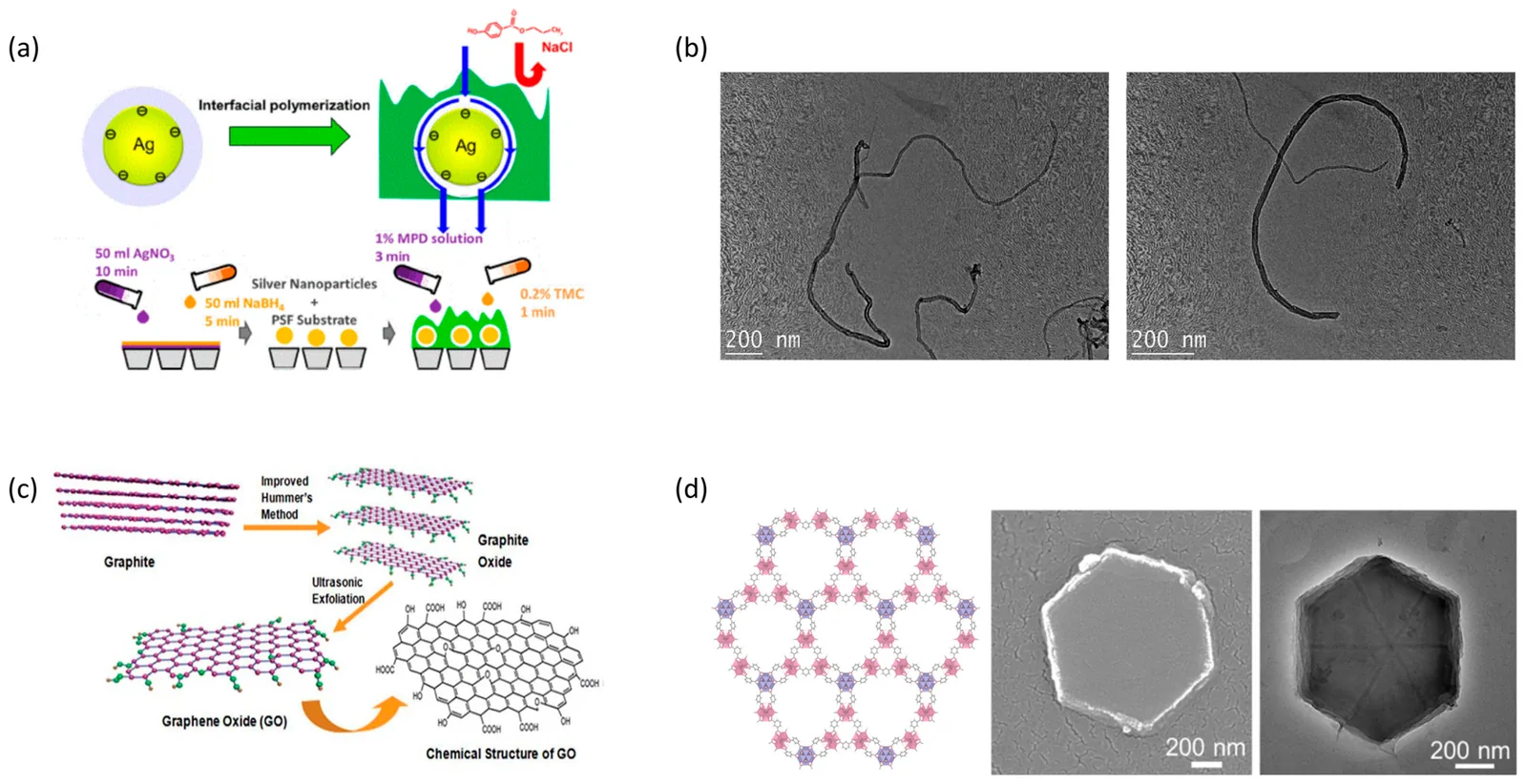
Structures of 0D, 1D, 2D, and 3D nanomaterials. (**a**) Silver nanoparticles were loaded on polysulfone (PSF) as the support substrate, and the antibacterial film was prepared by using the antibacterial property of silver [38]. (**b**) Reduced graphene oxide (TEM) images of pristine and functionalized multi-walled carbon nanotubes [69]. (**c**) Microparticle structure of graphene oxide (GO) [30]. (**d**) Structure of MOF nanomaterials [95].

AgNPs have attracted much attention for membrane modification because of their efficient and broad-spectrum bactericidal ability [38,39,40]. Their high specific surface area and surface reactivity endow membranes with significant antibacterial functions, mainly through the release of Ag^+^ and the generation of reactive oxygen species (ROS) [39,40,41]. AgNPs have been successfully loaded onto various substrates, including cellulose acetate, polylactic acid, polyvinyl alcohol, polyacrylonitrile, polyamide, and polysulfone [41,42,43]. Silver nanoparticles were loaded onto polysulfone (PSF) as a supporting substrate (as shown in Figure 1a). Particle size and surface chemical state are the core internal factors that determine their antibacterial efficacy. The smaller the particle size and the larger the specific surface area of silver nanoparticles, the faster the release rate of Ag^+^ and the higher the antibacterial activity [44,45]. However, small-sized particles can easily lead to encapsulation, resulting in a decrease in active sites. For example, Ag can be chemically anchored on the surface of GO to produce ~5 nm uniform particles, which can effectively overcome agglomeration and maintain high dispersion [46,47,48]. At the same time, the thickness and composition of the oxide layer on the surface of AgNPs directly regulate the release kinetics of Ag^+^ [24,39]. The spatial distribution determines the antibacterial efficiency of silver nanoparticles. When AgNPs are enriched on the membrane surface and directly exposed to the bacterial solution, their antibacterial effect is much better than that of AgNPs embedded in the matrix, which lose some contact activity due to polymer embedding [48,49]. For the above bottlenecks of agglomeration, burst release and burial, the current engineering strategies mainly focus on carrier loading (e.g., GO-Ag hybrid) or surface shell coating (e.g., Ag@RF), in order to realize the collaborative optimization of slow-release and long-acting antibacterial activity [40,46,49].

**Copper nanoparticles (CuNPs)**

With the advantages of broad-spectrum antimicrobial activity, environmental stability, low drug resistance, high catalytic activity and low cost, CuNPs have become a powerful substitute for AgNPs [50,51,52]. The particle size effect follows the same behavior as AgNPs. Smaller particles provide a larger specific surface area and faster Cu^2+^ release. If there is excessive release of copper ions, it can easily cause secondary pollution [52,53,54]. In addition, the composition of surface chemical states directly regulates the ion dissolution kinetics and bactericidal efficacy [50,51]. At present, CuNPs have been loaded on membrane materials such as polyether sulfone, polypropylene, polyacrylonitrile and polyamide by electrostatic adsorption, interfacial polymerization and non-solvent induced phase separation [55,56,57].

To solve the problem of agglomeration and burst release caused by particle size, researchers usually pre-fix CuNPs with dopamine, TiO_2_ or reduced graphene oxide carriers to improve dispersion and buffer initial burst release [58,59,60]. However, the carrier only solves the stability of the particles, and the spatial distribution in the membrane further determines the efficiency. Due to the polymer barrier, the release path of CuNPs embedded in the membrane matrix is extended and the rate is reduced, but some active sites are buried and inactivated [51,54]. On the contrary, the surface-loaded CuNPs are highly exposed and sterilize rapidly, but they face the risk of accelerated release due to direct contact with the liquid phase, which requires supplementation by Ag@RF or similar shell-coating strategies to achieve a balance.

#### 2.1.2. Metal Oxide Nanomaterials

**ZnO Nanomaterials**

ZnO nanomaterials have become a research hotspot due to their excellent antibacterial properties, good biocompatibility, and low cost [11,13,57]. Their antibacterial efficacy is highly dependent on particle size: decreasing size dramatically increases specific surface area and surface unsaturated atoms, thereby boosting surface reactivity [57,61]. Specifically, smaller ZnO nanoparticles provide more active sites for reactive oxygen species (ROS) generation and Zn^2+^ release, but excessive reduction in size may induce agglomeration, which reduces the effective exposed area [37,57].

Given these size-dependent antibacterial mechanisms, incorporating ZnO into polymer membranes has been explored as a promising strategy to enhance anti-biofouling performance [11,57]. Beyond its antibacterial function, ZnO also acts as a hydrophilic modifier: increasing ZnO loading progressively reduces the membrane contact angle, elevates porosity and average pore size, and consequently improves pure water flux [11,37,57]. The modified membranes exhibit potent antibacterial effects, primarily through the combined actions of ROS, released Zn^2+^, and direct physical damage to bacterial cell walls, achieving >99.9% inactivation against *E. coli* [37,61].

**TiO_2_ Nanomaterials**

TiO_2_ nanoparticles are another widely used metal oxide nanomaterial for membrane modification, owing to their excellent photocatalytic activity, chemical stability, low cost, and environmental friendliness [60,62,63]. Their particle size plays a critical role in performance; smaller TiO_2_ nanoparticles offer a higher specific surface area and more active sites, but simultaneously face a greater tendency to agglomerate, which diminishes light absorption and reactive oxygen species (ROS) generation efficiency [29,57,64]. Incorporating TiO_2_ into the polymer matrix significantly improves membrane hydrophilicity and antifouling performance [44,62,65]. For instance, in a cellulose acetate/polyurethane mixed matrix membrane, TiO_2_ modification notably reduced the water contact angle and increased pure water flux, while also conferring strong antibacterial activity against *E. coli* and *S. aureus* [62,64]. The antibacterial efficiency of TiO_2_ is highly dependent on light conditions. To extend its photoresponse into the visible range, doping with metal (e.g., Ag) or non-metals (N, F, S, C) elements has been widely adopted, significantly boosting the antibacterial efficiency under ambient or low-light conditions [62,63,64].

The antibacterial performance of 0D metal nanoparticles is highly dependent on their size and surface state. Smaller particles have a larger specific surface area and faster ion release rates, but they also tend to aggregate more readily. Therefore, the core design challenge for 0D materials is how to suppress aggregation and burst release without sacrificing antibacterial activity. Carrier loading and surface coating are two main strategies currently used to address this issue.

### 2.2. One-Dimensional Nanomaterials

#### 2.2.1. Nanotubes

Carbon nanotubes (CNTs) are known for their high aspect ratio, large specific surface area, high mechanical strength, and chemical stability [66,67,68]. CNTs with high aspect ratio are more likely to penetrate bacterial cell walls, and surface functionalization can enhance their interaction with cell membranes. Therefore, the diameter, length and surface functionalization of carbon nanotubes are crucial to their antibacterial activity [67,69,70]. According to the TEM image of carbon nanotubes, the structure of CNTs can be observed (as shown in Figure 1b). Single-walled carbon nanotubes (SWCNTs) are formed by the rolling of a single layer of graphene, with an extremely small diameter (typically <2 nm), making them more prone to penetrating cell walls when in contact with bacteria. Therefore, they often exhibit stronger bactericidal activity than multi-walled carbon nanotubes [67]. However, the extremely high surface energy of SWCNTs makes them more prone to agglomeration and more difficult to disperse in membrane matrices, limiting their application in certain high-flux membranes [68,69]. Multi-walled carbon nanotubes (MWCNTs) are formed by the rolling of multiple layers of graphene sheets, with stronger rigidity, relatively good dispersibility, and relatively low cost. They not only enhance the mechanical properties and hydrophilicity of the membrane, but also reduce the surface roughness of the membrane to minimize initial adhesion sites for bacteria [68,69]. Compared to SWCNTs, which focus on direct killing, MWCNTs are more inclined to provide structural stability and an anti-adhesion surface [70,71,72].

In membrane modification, CNT-PA nanocomposite reverse osmosis membranes (RO) have shown lower surface roughness and higher hydrophilicity [70]. CNTs act as stabilizers during the interfacial polymerization process, are evenly distributed at the polymer interface, and reduce polymer agglomeration. This process effectively inhibits the generation of non-selective defects in the polyamide selective layer, ensuring that the membrane maintains excellent salt rejection performance while enhancing water flux. Furthermore, due to the extremely high mechanical strength of CNTs, their introduction significantly enhances the rigidity of the polyamide layer, making the membrane exhibit stronger compressive resistance under high-pressure operating conditions, thus avoiding performance degradation caused by densification of the membrane structure [70].

Halloysite nanotubes (HNTs), as a natural aluminosilicate clay mineral, exhibit distinct functions from the former two. HNTs themselves do not possess potent inherent antibacterial activity. Instead, their tubular cavities are utilized as containers to load and slowly release silver ions or antibacterial agents [66,73]. Furthermore, the abundant hydroxyl groups on the surface of HNTs endow them with excellent hydrophilicity, often serving as horizontally arranged hydrophilic interlayers to reduce water transport resistance. Therefore, the core contributions of HNTs in membrane modification lie in improving hydrophilicity and functioning as a drug carrier, rather than direct physical puncture for sterilization [66,73].

#### 2.2.2. Nanofibers

Nanofiber membranes prepared by electrospinning have the advantages of large specific surface area, high porosity, and controllable fiber diameter [55,61]. For example, loading AgNPs into hyperbranched polyethyleneimine (HPEI) nanofibers yields membranes with both antibacterial and antifouling functions. In terms of antifouling, the flux recovery ratio (FRR) of the modified membrane increased significantly from 51.8% for the pristine polyethersulfone (PES) membrane to values between 68.43% and 86.71% [27,46].

The advantages of one-dimensional nanomaterials in membrane modification come from their anisotropic shape. In practice, however, these materials tend to aggregate, which reduces the antibacterial effect that their geometry could otherwise provide. Therefore, the key to their application is not simply having a high aspect ratio, but rather using surface functionalization to improve dispersion and to guide favorable orientation and exposure. Only in this way can the geometric benefits be truly translated into enhanced membrane performance [66,74].

### 2.3. Two-Dimensional Nanomaterials

2D nanomaterials are layered materials with only one dimension at the nanoscale, including graphene oxide, carbon nitride, molybdenum disulfide, and others [75,76,77]. Their high specific surface area and tunable surface chemistry make them ideal candidates for membrane modification. Unlike the linear structure of 1D materials, the sheet-like structure of 2D materials not only provides an enormous specific surface area but also enables physical disruption of cell membranes through their edges [76,78].

#### 2.3.1. Graphene Oxide (GO)

GO is an oxidized form of graphene, which can be prepared by chemical oxidation at a relatively low cost [63,75]. GO has both a large specific surface area and hydrophilicity, the latter originating from oxygen-containing functional groups such as carboxyl, epoxy, and hydroxyl groups. It is widely used in nanocomposite membranes [46,48]. The lamellar structure, lateral size, concentration and number of layers of GO significantly affect its performance [57,75]. Smaller sheets of GO are easier to pass through the bacterial cell membrane, and the thickness regulates its contact area with bacteria [76,78]. The particle structure of GO is shown in Figure 1c. To enhance functionality, GO is often combined with metal nanoparticles, photocatalysts, polymers, and bactericidal compounds [48,49,57]. GO nanosheets can either disperse nanoparticles on their structure or act as molecular sieves, allowing particles smaller than their interlayer spacing to pass through [75,76]. The location of GO in the membrane has a great impact on antibacterial efficiency. Surface-coated GO sheets are directly exposed to the bacterial solution and show much higher antibacterial activity than GO blended in the matrix, where it is wrapped by polymer.

#### 2.3.2. Carbon Nitride Nanomaterials

Graphitic carbon nitride (g-C_3_N_4_) [79] nanosheets have a layered structure with regularly distributed triangular nanopores. Their lattice defects are about 3.1 Å, and they are stable in aqueous solution over a wide pH range, serving as efficient water transport channels [80,81,82]. Using tannic acid (TA) functionalized g-C_3_N_4_ nanosheets to construct an interlayer can form a highly hydrophilic layer with low water transport resistance and strong structural stability [79,81]. In the synergistic application of g-C_3_N_4_ nanosheets and HNTs, the 2D nanosheets provide molecular sieve channels, while the one-dimensional nanotubes enhance hydrophilicity and mechanical stability, which fully reflects the advantages of the combination of nanomaterials with different dimensions [73,83].

#### 2.3.3. Molybdenum Disulfide Nanomaterials

MoS_2_ nanosheets possess a high specific surface area and sharp edges, which form the basis for their ability to disrupt bacterial cell membranes through physical contact. Since fewer layers and more fully exposed edges lead to more significant bactericidal effects, the antibacterial efficiency depends on the layer number and edge exposure degree [84,85,86]. To fully leverage this advantage, polydopamine (PDA) modification effectively inhibits the agglomeration of MoS_2_ in the polyamide matrix, improves its dispersibility, ensures adequate exposure of active sites on sharp edges, and prevents the loss of piercing ability due to excessive polymer embedding [84,85]. Meanwhile, the self-polymerization characteristics of PDA contribute to the formation of defect-free thin-film nanocomposite (TFN) membranes during interfacial polymerization, endowing the membranes with excellent salt rejection and high compressive strength. Additionally, functional groups (e.g., hydroxyl and amino) introduced by PDA significantly enhance membrane surface hydrophilicity and negative charge repulsion [84,85]. The antibacterial and anti-pollution properties of the membrane are jointly enhanced through the synergistic effect of physical cutting action and the physicochemical properties of the membrane surface [84,85,86].

#### 2.3.4. MXene Nanosheets

MXene is a new type of 2D nanomaterial, which is composed of transition metal carbides or nitrides. The general chemical formula can be expressed as M_n+1_X_n_T_x_. MXene nanosheets have attracted wide attention in membrane modification due to their unique layered structure, excellent electrical conductivity, high specific surface area, and good hydrophilicity [1,77].

The antibacterial activity of MXene-modified membranes originates from multiple synergistic mechanisms. First, the sharp edges of MXene nanosheets can puncture bacterial cell membranes via physical contact, causing leakage of intracellular contents—similar to GO and MoS_2_ nanosheets [87,88]. Second, the abundant functional groups (e.g., -OH, -O) on MXene surfaces impart some chemical bactericidal activity through oxidative stress [1,89]. In addition, the excellent photothermal conversion and electrical conductivity of MXene enable local heating or electroporation under near-infrared light or a weak electric field, significantly enhancing bactericidal efficiency [1].

MXene nanosheets can also be incorporated as nanofillers into polymer matrices to prepare TFN membranes. Their 2D layered structure provides fast water transport channels while improving surface hydrophilicity and antifouling performance [77,90,91]. The incorporation of functionalized MXene nanosheets into ceramic hollow fiber NF membranes demonstrates the potential of MXene in complex water treatment scenarios [77,89]. The orientation and interlayer spacing of MXene nanosheets in the membrane are key factors determining separation performance and antibacterial efficiency [77,89].

The research value of 2D material membranes lies in their dual functions: antibacterial activity and the provision of water transport channels. However, these functions are not inherently achieved; they strongly depend on the orientation, interlayer spacing, and exposure state of the materials within the membrane. Specifically, the edges must be fully exposed on the surface to physically kill bacteria, and the interlayer channels need to be properly oriented to enable fast water permeation [82,89]. Therefore, precise control of these structural parameters is a top priority in this research field.

### 2.4. Three-Dimensional Nanomaterials

The core characteristic of three-dimensional nanomaterials is that they possess an ordered internal structure or pore system that spans three-dimensional space and is at the nanoscale [91,92,93]. In this review, we define such materials as functional materials with a three-dimensional nanoscale internal structure, and metal–organic frameworks (MOFs) are typical representatives, so this section will only discuss MOFs.

MOFs are constructed from metal nodes and organic linkers, forming periodic nanopores, and they feature uniformly tunable pore structures, high specific surface areas, and chemical functionalization capabilities [32,94]. The structure of MOF nanomaterials is shown in Figure 1d. MOFs are usually partially embedded in the polymer matrix, and their antibacterial activity mainly relies on the internal pores, pore topology, and metal node composition. Smaller pore sizes can extend the ion diffusion path for sustained release, while different metal nodes provide different activities and release kinetics [91,92,95]. In addition, particle size and lattice defects affect dispersibility and interfacial compatibility; particles that are too small tend to agglomerate, while those that are too large may create non-selective defects [70,96]. Compared with zero-dimensional metal nanoparticles, the three-dimensional pore channels of MOFs significantly prolong the release path of metal ions, achieving long-term release [91,92,95]; furthermore, the organic ligands released upon degradation of MOFs may have synergistic antibacterial effects [32,34,91]. In specific applications, MIL-101(Fe), a 3D nanomaterial with a rich porous topology [70,96], can significantly increase water flux through its multi-channel structure and can also be enriched on the membrane surface via electrostatic surface segregation [9,70,96]. Silver-containing MOFs consist of metal nodes and organic linkers, allowing precise tuning of antibacterial performance through the choice of metal and ligand [97,98,99]. Ag-MOFs are often grafted onto membrane surfaces via ultraviolet (UV)-initiated polymerization [98].

While 0D and 1D materials can directly interact with bacteria when exposed on the membrane surface, 3D MOFs are usually wrapped by polymer in the matrix. Their antibacterial action mainly relies on the slow release of bactericidal metal ions through ligand exchange or hydrolysis of metal nodes, rather than on the complete degradation of the material [91,92]. The advantage of this release mode is the prolonged release path due to the physical confinement of the pore structure. However, the release rate is still limited by factors such as MOF framework stability, water permeation, and ligand exchange kinetics [61,95].

### 2.5. Summary of Key Structures and Performance Relationships of Nanomaterials Across Various Dimensions

Based on the above discussion, the antibacterial activity of nanomaterials with different dimensions is not solely determined by the dimensions themselves, but is jointly regulated by various factors such as material structural parameters, surface chemical states, position within the membrane, and degree of exposure. Table 1 systematically summarizes the key structural variables, in-membrane states, interfacial properties, as well as the contributions and limitations of representative nanomaterials with different dimensions to antibacterial and separation performance.

## 3. Preparation Methods of Nanomaterial-Modified Membranes

Membranes themselves often lack sufficient antibacterial ability, and their surfaces tend to become substrates for microbial attachment and proliferation during long-term operation [3,8,10]. Microbial growth on the membrane surface not only reduces flux but also accelerates irreversible fouling [5,7,12]. The introduction of nanomaterials into the membrane structure aims to utilize the inherent bactericidal or bacteriostatic activity of nanomaterials to endow the membrane surface with the ability to actively resist microbial attacks [101,102]. The essence of this modification is to prevent microorganisms from attaching and to enable the membrane to actively resist them. Incorporating functionalized nanomaterials into the membrane structure is a key technical step for preparing long-lasting antibacterial membranes. An ideal antibacterial membrane should have high loading and high exposure of antibacterial agents on the substrate, while controlling the release of antibacterial components [14,102]. Currently, surface coating, surface grafting, and matrix blending are the three main incorporation strategies adopted [11,98,99].

### 3.1. Surface Coating

Surface coating can be achieved in two ways. One is to directly coat the antibacterial nanomaterials onto the membrane surface, and the other is to immerse the membrane in a solution containing antibacterial nanomaterials [51,99]. Both methods rely on interactions such as hydrogen bonding and crosslinking to introduce nanomaterials onto the membrane surface, ultimately reducing bacterial adhesion and inhibiting biological pollution.

For example, an antibacterial membrane was prepared by coating a polyethersulfone surface with a chitosan/polyphenol solution followed by crosslinking with glutaraldehyde. This membrane belongs to the ultrafiltration (UF) category, with the chitosan/polyphenol coating located on the membrane surface. When the chitosan content is 1.0 wt%, the antibacterial zone diameter of the membrane against *Escherichia coli* is 7.83 mm, the antibacterial efficiency reaches 99.0%, and porosity decreases from 59.6% to 44.5%, reflecting the growth inhibitory effect of the modified membrane [103]. As adsorption time increased, coating density rose, causing the pure water permeability to drop from 31.42 to 9.37 L·m^−2^·h^−1^·bar^−1^, and the average pore size to shrink from 2.11 nm to 1.10 nm. This indicates that while thickening the coating enhances antibacterial performance, it comes at the cost of permeate flux and significant pore blockage. Nevertheless, this dense coating layer did not introduce obvious defects; instead, it improved the retention capacity for small-molecular pollutants [103].

An alternative coating approach involves inorganic nanosheets. For instance, MXene nanosheets were deposited onto a ceramic ultrafiltration membrane substrate via pressure-assisted filtration, where dehydration condensation between MXene and the ceramic surface formed chemical bonds, reducing pore size from approximately 2.9 nm to 1.2 nm [90]. Compared with organic coatings relying on physical crosslinking, the chemically bonded MXene coating has better stability [90].

**Figure 2:**
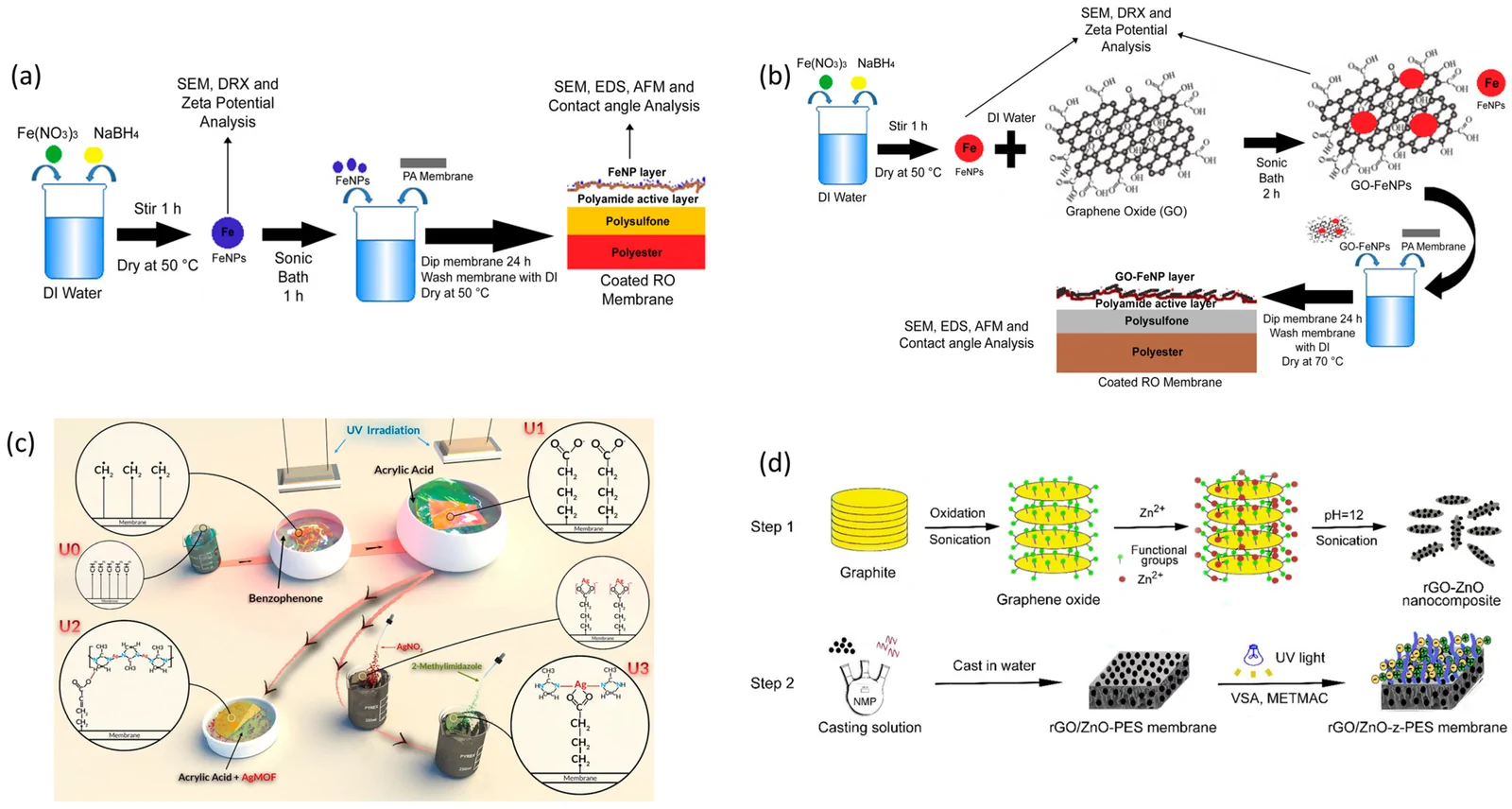
Preparation methods of nanomaterial-modified membranes. (**a**,**b**) Preparation process of FeNPs and GO-FeNPs coated on reverse osmosis membrane [99]. (**c**) Surface modification process of Ag-MOFs grafted membrane [98]. (**d**) Operation process of rGO/ZnO-z-PES bifunctional membrane obtained by blending [11].

In terms of inorganic nanosheet coating, the method of coating iron nanoparticles (the coating process is shown in Figure 2a) and graphene oxide–iron nanoparticle composites (the coating process is shown in Figure 2b) on commercial polyamide reverse osmosis membranes exhibits unique advantages in anti-biofouling compared to organic coatings and inorganic MXene coatings. The research results indicate that graphene oxide–iron nanoparticle composites (GO-FeNPs) coating have a lower tendency for agglomeration and surface roughness compared to pure iron nanoparticles [99].

Surface coating positions nanomaterials on the membrane surface, offering advantages such as a simple preparation process and sufficient exposure of antibacterial sites. However, the reproducibility of this method is susceptible to coating uniformity, and uneven coatings may lead to membrane defects. An excessively thick coating layer can block membrane pores, significantly reducing permeation flux, while an excessively thin layer may fail to form a continuous antibacterial layer. Since the coating layer is mainly bound to the substrate through physical interactions such as hydrogen bonding or van der Waals forces, there is a risk of detachment during long-term operation and repeated cleaning, indicating a need for improved stability [10,14].

### 3.2. Surface Grafting

Compared with surface coating, surface grafting first forms active sites on the substrate surface to initiate graft polymerization, and then covalently grafts antibacterial nanomaterials onto the substrate, thereby effectively improving the antibacterial ability of the membrane [27,46,61]. Since some antibacterial nanomaterials or polymers are chemically inert, pretreatment such as graft polymerization, plasma treatment, alkali treatment, ATRP, or photocatalysis is usually needed to introduce reactive functional groups onto the membrane surface [98,104].

Atom transfer radical polymerization (ATRP) is widely adopted due to its broad monomer compatibility, flexible polymerization methods, and mild reaction conditions. Its core mechanism involves the formation of carbon–carbon bonds through atom transfer radical addition catalyzed by transition metals [104,105]. Studies show that grafting polyacrylamide (PAAm) onto polyamide thin-film composite reverse osmosis membranes via ATRP locates the PAAm polymer within the graft layer on the membrane surface. Fluorescence counting of green (live) and red (dead) bacterial cells revealed that the grafted RO membrane exhibited a dead-to-live cell ratio of approximately 27, which was 650-fold higher than that of the unmodified membrane. Meanwhile, the antibacterial rates against *Escherichia coli* and *Bacillus subtilis* increased from 90.6% and 91.9% to 98.8% and 99.0% [104]. After modification, the flux recovery ratio (FRR) increased from 95.3% to 97.6%, and the contact angle decreased from 35.3° to 31.6° [104]. Furthermore, UV-mediated grafting technology is also an efficient and irreversible surface functionalization strategy [98]. Acrylic acid (AA) was grafted onto the surface of polysulfone (PSf) ultrafiltration membrane through UV-induced graft polymerization, followed by the introduction of silver-containing metal–organic frameworks (Ag-MOFs) to endow the membrane with antibacterial properties. Following contact of the bacterial suspension with the membrane surface, staining and enumeration showed that the grafted membrane achieved inactivation rates of 90% for *E. coli* and 95% for *S. aureus*. The silver ion release experiment (as shown in Figure 2c) showed that the original growth sample had a cumulative silver release concentration of only 0.2 mg/L after 15 days of testing, and the release rate was stable and lower than that of other samples, confirming the long-term antibacterial potential of Ag-MOFs grown in situ [98].

Unlike physically bound coatings, grafting firmly anchors nanomaterials onto the membrane surface through covalent bonds, offering superior binding stability, full exposure of antibacterial sites, and high antibacterial efficiency. However, this method involves complex procedures, often requiring activation pretreatment of the membrane surface, and the grafting density is difficult to precisely control, making reproducibility challenging. Excessive grafting density may increase mass transfer resistance, leading to a decline in permeation flux [61,106]. Although covalent bonding theoretically provides good durability, under harsh chemical cleaning conditions, the bond strength between the graft layer and the membrane substrate still requires long-term verification, and maximum durability cannot be guaranteed under all operating conditions [27,46,107].

### 3.3. Matrix Blending

Matrix blending involves physically dispersing nanomaterials in a polymer solution, followed by phase inversion or solvent evaporation to form the membrane. This is the simplest method for preparing antibacterial membranes [58,69,91]. The addition of nanomaterials can change the surface roughness, water contact angle, pore size and porosity, thereby affecting permeation, antibacterial, and antifouling properties [11,108].

For example, the reduced graphene oxide–zinc oxide nanocomposite material (rGO/ZnO) was blended into a polyether sulfone (PES) membrane matrix, and the rGO/ZnO-PES blend membrane was prepared by the non-solvent induced phase separation method. The rGO/ZnO-z-PES bifunctional membrane was prepared by UV-induced graft polymerization (as shown in Figure 2d). In terms of filtration performance, the rGO/ZnO blend increased the pure water permeability of the membrane from approximately 323 L·m^−2^·h^−1^·bar^−1^ to 641 L·m^−2^·h^−1^·bar^−1^, and the resulting rGO/ZnO-PES membrane exhibited a molecular weight cut-off of 280 kDa, which still falls within the ultrafiltration range. Dynamic filtration experiments showed that in the 8-h humus organic pollution experiment, the increase in transmembrane pressure (TMP) of the rGO/ZnO-z-PES membrane was much smaller than that of the original PES membrane and rGO/ZnO blend membrane [11]. Dynamic filtration tests showed permeate zinc ion concentration approximately 0.17 mg/L, below irrigation standard. After 3 h of membrane–bacteria contact, plate counting revealed that bactericidal efficiency of the rGO/ZnO-PES membrane increased with nanocomposite fraction, corresponding to decreased viable counts of *E. coli* and *B. subtilis* [11]. In another example, Ag@RF nanofillers were uniformly dispersed in aqueous piperazine solution and participated in the formation of the PA selective layer via interfacial polymerization (IP). This membrane belongs to the nanofiltration (NF) category, and Ag@RF is located at the doping site of the selective layer. At 0.02 wt% doping, Ag@RF formed irregular granular structures that increased the permeation area. At 0.03 wt%, agglomeration and oversized nodules caused simultaneous declines in both flux and rejection rate. The abundant hydroxyl groups on the Ag@RF surface reacted with TMC to form ester bonds, enhancing the binding between nanofillers and the PA layer [29,40]. The water flux of the membrane increased from 80 to 150 L·m^−2^·h^−1^. Due to the special core–shell structure of Ag@RF, this membrane also exhibits the expected Ag^+^ slow-release capability, which is beneficial for long-term resistance to biofouling. Plate count assays demonstrated that the modified membrane achieved an *E. coli* inactivation rate of 99.6% [40]. The particle leaching issue is effectively controlled by the physical barrier effect of the core–shell structure.

Matrix blending embeds nanomaterials within the membrane matrix or selective layers, featuring a simple preparation process and ease of scale-up for production [8,69,91]. However, the dispersibility and compatibility of nanomaterials in the polymer matrix pose key challenges, as they are prone to agglomeration, leading to non-selective defects or pore blockage within the membrane. Nanomaterials located deep within the matrix are encapsulated by the polymer, resulting in insufficient exposure of antibacterial sites and reduced activity utilization [8,58]. Simultaneously, during long-term filtration processes, there is a risk of leaching of nanoparticles or metal ions, which may affect the long-term stability of the membrane and water quality safety [10,27].

### 3.4. Summary of Nanomaterial Immobilization Strategies

The immobilization of nanomaterials is crucial for the application of antimicrobial films, as it effectively balances antimicrobial performance and safety, preventing the leaching of nanoparticles or metal ions during operation, thereby avoiding secondary pollution and rapid decay of antimicrobial efficacy. Currently, the main immobilization strategies encompass various methods such as chemical grafting, silica or polymer coating, core–shell structure design, surface functionalization, and carrier loading or matrix blending [11,98,99].

Different immobilization strategies exhibit varying performance in terms of binding method, advantages and disadvantages, and long-term stability (as shown in Table 2). Improvements in the future could focus on multi-strategy synergistic immobilization schemes that balance the simplicity of membrane preparation with long-term stability.

## 4. Antibacterial Mechanisms, Synergistic Effects, and Target Bacteria of Nanomaterial-Modified Membranes

Coating, grafting, and blending are three common strategies for introducing nanomaterials into membranes, all sharing the same ultimate objective of enabling the membrane surface to actively fend off microorganisms. The remarkable antibacterial efficacy of these modified membranes stems from the synergy between the nanomaterials and the membrane substrate. On one hand, the membrane acts as a support that distributes and secures the nanomaterials. On the other hand, the nanomaterials function as the killing agents, operating through multiple physicochemical routes [33], predominantly including metal ion leaching, ROS generation, physical membrane puncture, and surface anti-adhesion.

### 4.1. Antibacterial Mechanisms of Nanomaterial-Modified Membranes

#### 4.1.1. Metal Ion Release Bactericidal Mechanism

Antibacterial action in membranes modified with metal and metal oxide nanoparticles such as AgNPs and CuNPs is mainly accomplished via ion release [38,39,43].

**Figure 3:**
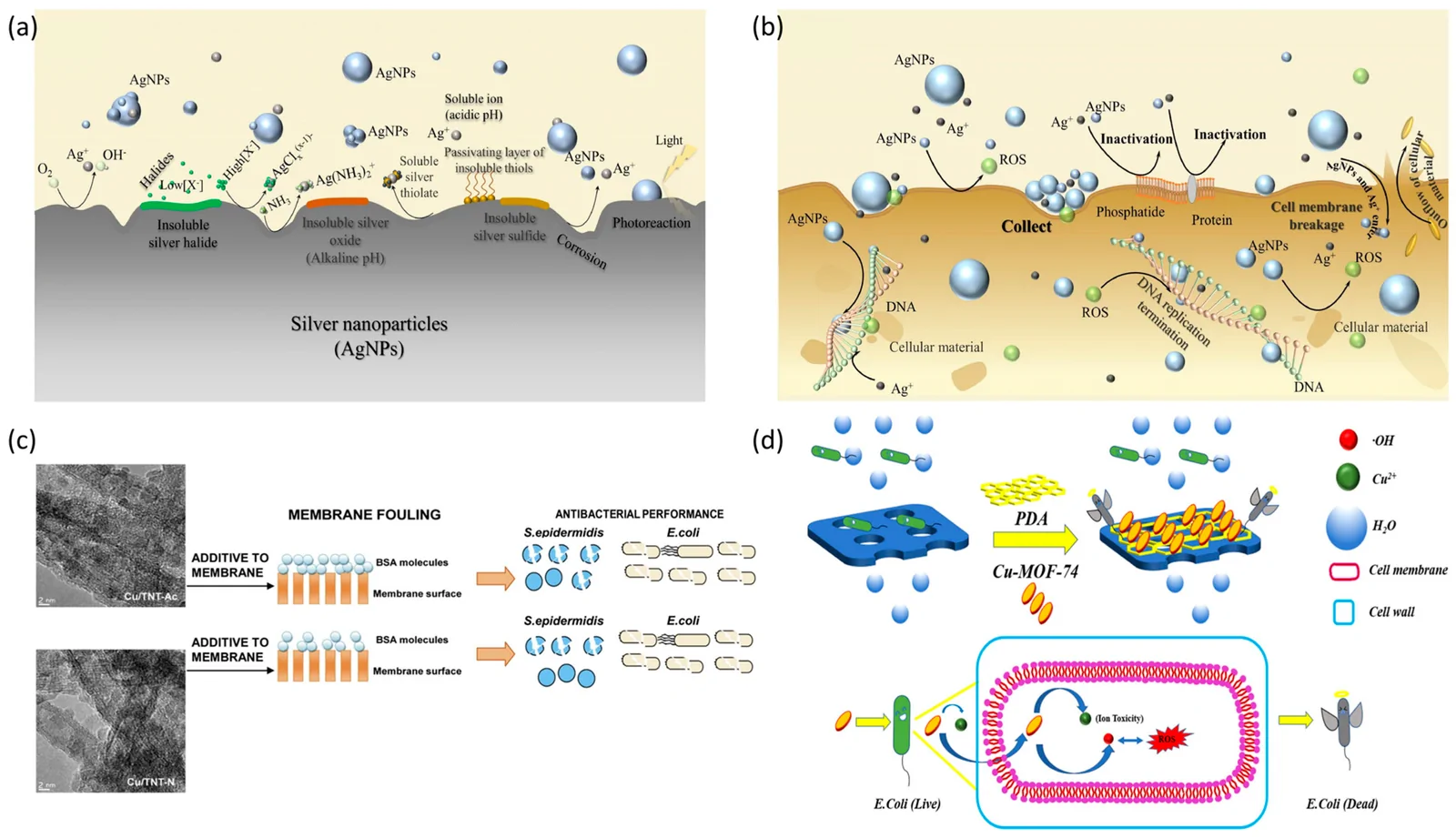
Metal ion releases bactericidal mechanism. (**a**,**b**) The release mechanism of silver ion nanoparticles [39]. (**c**,**d**) The release and sterilization mechanism of copper ion nanoparticles [50,97].

For AgNPs-modified membranes, the process involves the gradual oxidation of AgNPs and the release of Ag^+^ ions (as shown in Figure 3a). The released Ag^+^ penetrates the bacterial cell wall (as shown in Figure 3b) and coordinates with the sulfhydryl (-SH) groups in enzyme proteins, leading to the inactivation of enzymes [37,38]. Key evidence supporting this mechanism comes from ion control experiments. For example, studies have shown that the antibacterial activity of AgNPs-containing membranes is almost identical to that of solutions containing the same concentration of free Ag^+^, confirming that the released ions are the primary bactericidal agents [39,78]. In GO-Ag composite membranes, the GO layers serve as carriers for AgNPs, and their own antibacterial activity synergizes with the Ag^+^ enhancement effect [47,48,49]. To prevent excessive release, researchers have designed core–shell structures (such as Ag@RF), and the ion release kinetics confirmed that the phenolic resin shell effectively regulated the release rate of silver ions, which was about 2.04 times lower than that of pure silver nanoparticles, indicating controlled and slow release [40]. The World Health Organization stipulates that the concentration of Ag^+^ in drinking water should not exceed 0.10 mg/L [39,78]. Proper membrane design can control the Ag^+^ release rate within the range of 0.0018–0.01 μg·cm^−2^·d^−1^, thus ensuring antibacterial activity while avoiding secondary pollution [39,78].

The antibacterial mechanism of CuNPs-modified membranes involves both membrane depolarization (as shown in Figure 3c) and ROS generation (as shown in Figure 3d) [28,34,37]. Membrane depolarization is considered the main pathway. Normal microbial cells maintain a transmembrane potential difference of about 100–200 mV. When Cu^2+^ released from CuNPs contacts the bacterial cell membrane, this potential difference drops sharply, leading to depolarization and eventual microbial death [28,37,39]. Ionic control experiments have shown that Cu^2+^ solutions of the same concentration may reproduce the antibacterial effect of CuNPs-modified membranes [37]. The release of Cu^2+^ is regulated by concentration, temperature, and pH. In membrane modification, CuNPs need to be pre-immobilized on a carrier (such as polydopamine) to inhibit excessive ion leaching and prevent secondary pollution [27,50,51].

#### 4.1.2. Reactive Oxygen Species (ROS) Oxidation Mechanism

**Figure 4:**
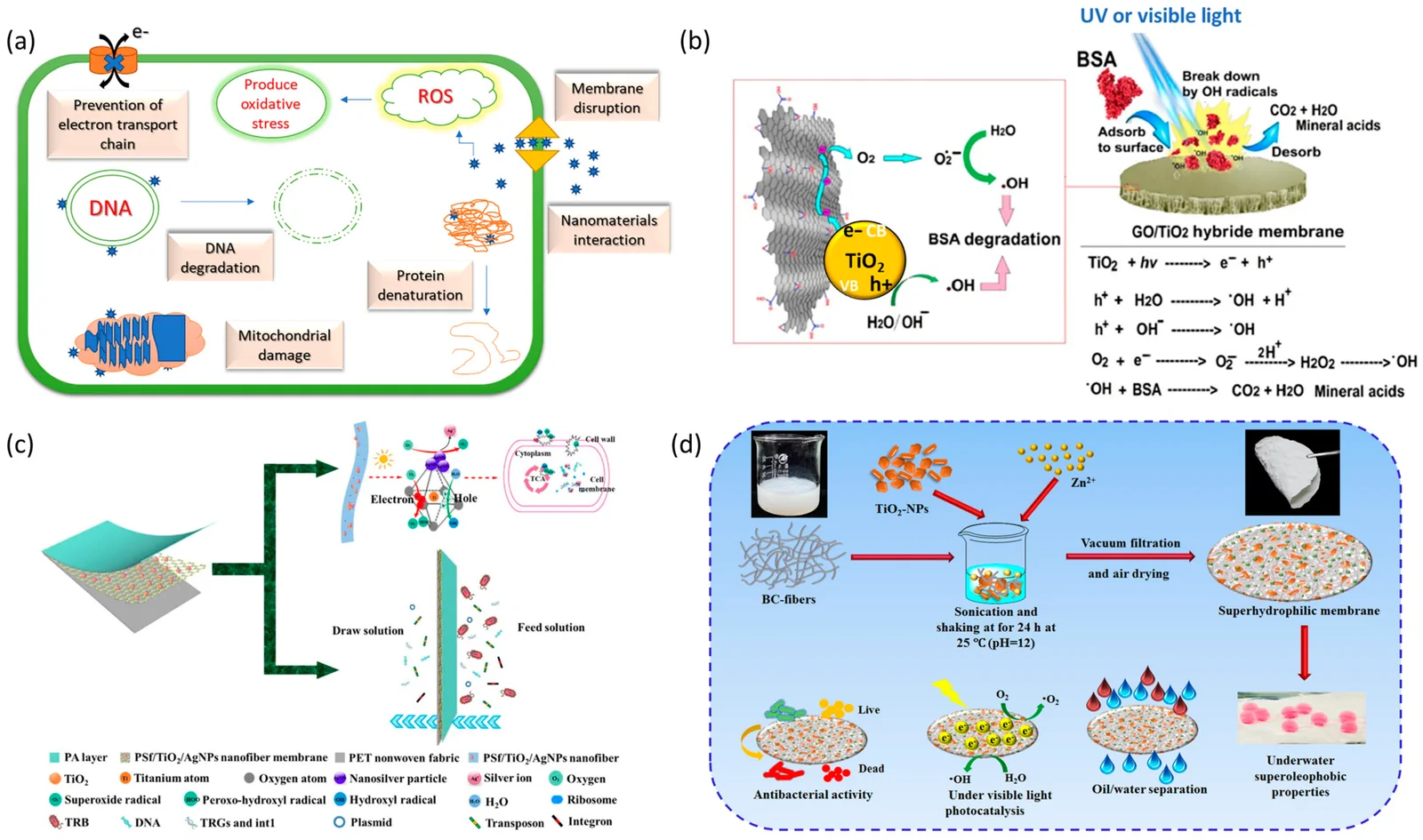
Reactive oxygen species (ROS) oxidation mechanism. (**a**) The interaction process of metal based nanomaterials with bacteria [13]. (**b**) The sterilization mechanism of titanium dioxide through ROS oxidation and the release of reactive oxygen species [63]. (**c**) Synergistic ROS-based bactericidal mechanism of Ag and titanium dioxide [64]. (**d**) The preparation process of zinc ions, TiO_2_, and hydrophilic membranes, which achieves sterilization effect through ROS release [60].

ROS generation is an important antibacterial pathway for metal oxide nanoparticle-modified membranes and some carbon-based nanomaterial-modified membranes [58,68,109]. The core mechanism is that nanomaterials on or near the membrane surface, under catalysis or photoexcitation, produce highly reactive species such as superoxide anion (O_2_^−^), hydrogen peroxide (H_2_O_2_), and hydroxyl radicals (·OH). These strong oxidants indiscriminately oxidize bacterial lipids, proteins, and nucleic acids attached to the membrane surface, ultimately causing cell death (as shown in Figure 4a) [58,78,109].

The antibacterial activity of TiO_2_ nanoparticle-modified membranes strictly depends on photocatalytic conditions [55,62,64]. When TiO_2_ is excited by ultraviolet light, electron-hole pairs are generated, which react with water and oxygen to produce ROS (as shown in Figure 4b) [55,63,64]. Light-dark control experiments confirm that the antibacterial activity of TiO_2_-modified films under UV or visible light irradiation is significantly higher than under dark conditions, confirming that light-dependent ROS generation is the dominant mechanism [21,39,50]. Doping with metals (e.g., Ag) (as shown in Figure 4c) or non-metals (N, F, S, C) can extend its light response to the visible region, improving efficiency under practical illumination [51,62,63]. In addition, a composite film was prepared by blending bacterial cellulose (BC) nanofibers with TiO_2_ nanoparticles and further in situ growth of ZnO nanoparticles. The synergistic effect of ZnO and TiO_2_ in the composite film not only enhances the visible light photocatalytic activity, but also achieves efficient sterilization by releasing zinc ions and generating reactive oxygen species (as shown in Figure 4d).

The antibacterial activity of ZnO-modified films may stem from the combined effects of Zn^2+^ dissolution, ROS generation, and direct ZnO–cell physical interactions, with the dominant pathway depending on pH, illumination, and water composition [37,58,59]. The involvement of ROS has been confirmed through scavenger experiments. Addition of ROS scavengers such as vitamin C or catalase significantly reduces the antibacterial efficiency of ZnO-modified films, confirming that ROS (e.g., H_2_O_2_) are key mediators of bacterial inactivation [59]. Ion exclusion experiments further show that treating bacteria with Zn^2+^ solutions of the same concentration as the film leachate exhibits almost no antibacterial activity, indicating that the primary bactericidal mechanism of this system is ROS-dependent rather than ion-dependent [58]. At neutral pH, the ion leaching rate of ZnO is much lower than that of AgNPs, so most water treatment scenarios will not cause obvious secondary metal ion pollution; however, under strongly acidic or alkaline feed conditions, Zn^2+^ leaching is non-negligible, and environmental safety must be assessed based on actual water quality [58].

#### 4.1.3. Physical Contact Disruption Mechanism

Physical contact disruption is a unique antibacterial mode for carbon-based and some 2D nanomaterial-modified membranes. Since its bactericidal action does not depend on chemical dissolution or photoexcitation, it offers environmental friendliness and long-term stability [23,75].

**Figure 5:**
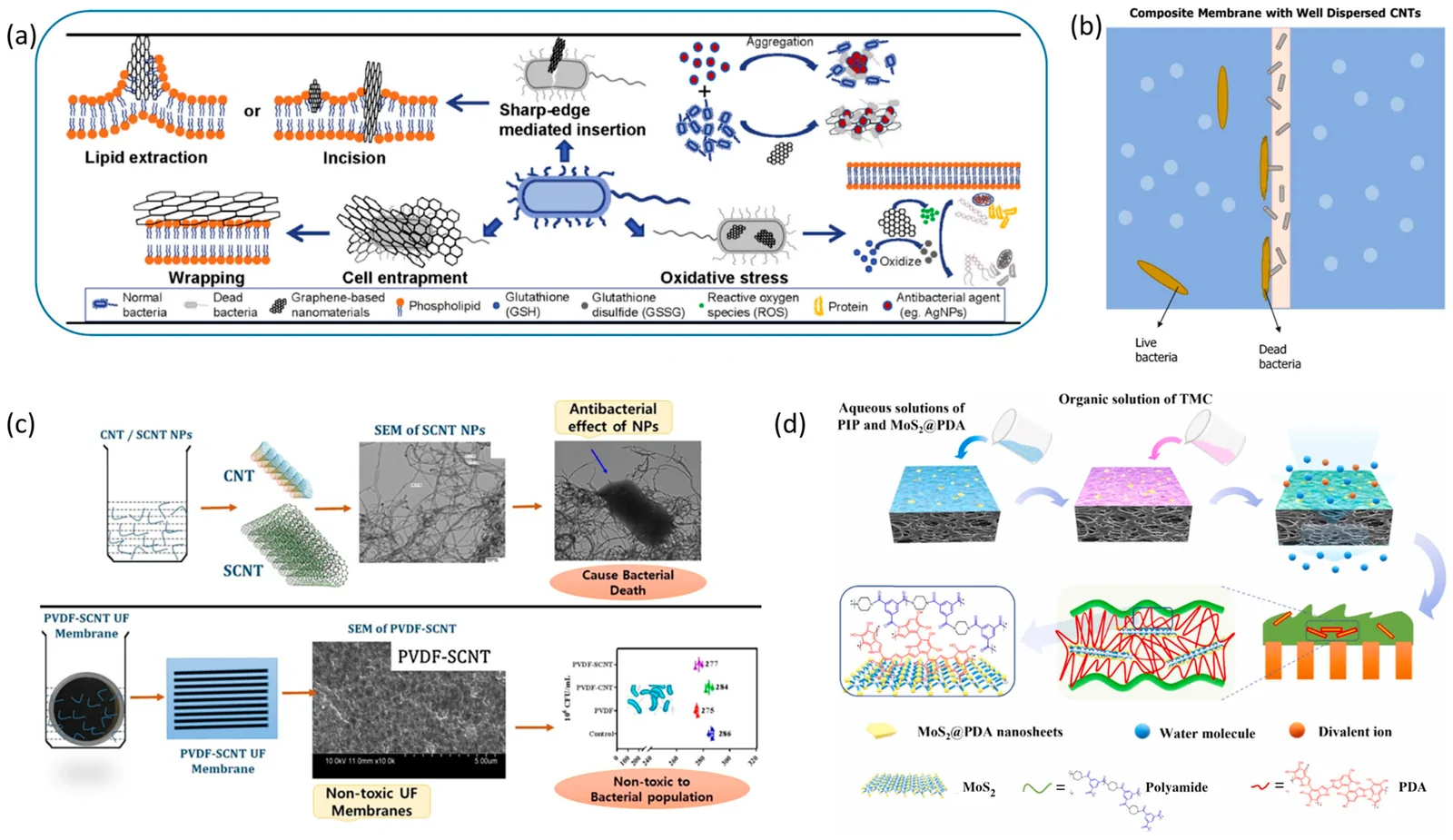
Physical contact disruption mechanism. (**a**) Various antibacterial mechanisms of GO, mainly including physical contact sterilization [76]. (**b**,**c**) Sterilization schematic diagram of CNT-modified polyvinylidene fluoride (PVDF) through physical contact rupture and preparation process of modified membrane [69,110]. (**d**) Simplified preparation process of MoS_2_ and physical contact rupture sterilization schematic [76].

The antibacterial activity of GO-modified membranes arises from the synergy between physical cutting and chemical oxidation (as shown in Figure 5a) [75,78]. The sharp edges of GO sheets exposed on the membrane surface produce mechanical stress when interacting with attached bacteria, piercing the cells and disrupting membrane integrity, leading to leakage of intracellular contents [76,78]. The GO membrane showed water permeability 1–2 orders of magnitude greater than commercial UF membranes. At the same time, oxidative stress induced by the oxygen-containing functional groups on GO further damages the cell membrane and internal components [75,78]. Scanning electron microscope (SEM) images show that *E. coli* cells treated with GO-Ag are severely damaged, with extensive destruction of the cell wall and membrane [28,46]. The transverse size, concentration, and exposure of GO sheets on the membrane surface affect the antibacterial efficiency of the GO-modified membrane. Among them, smaller GO sheets are more likely to penetrate the cell membrane, and the degree of enrichment of GO on the membrane surface directly determines the number of exposed antibacterial sites [28,46]. When GO is blended into the matrix, some sheets are wrapped by polymer and cannot contact bacteria, so the antibacterial efficiency is generally lower than that of surface-grafted or coated membranes [46,49,75].

The antibacterial activity of CNT-modified membranes manifests as a sequential physical disruption process [64,67,69]. First, the exposed CNTs on the membrane surface, with their extremely small diameter and high specific surface area, can penetrate the cell wall and membrane of attached bacteria, directly contacting the bacterial interior (as shown in Figure 5b). Second, CNT insertion disturbs the cell membrane structure, alters membrane potential, increases permeability, and causes leakage of cellular contents. Concurrently, local electron transfer induced by CNT insertion triggers oxidative stress, further exacerbating biomolecular damage (as shown in Figure 5c) [64,67,69]. Filtration, culture, and staining of *E. coli* showed no colonies on the CNT-modified membrane. It reduced *P. aeruginosa* biofilm formation by 87% and 92% at 24 h and 96 h, respectively. Compared to the pristine membrane, it exhibited an approximately 8-fold increase in flux, a reduced contact angle, and a 32% increase in tensile strength [69]. The anti-biofouling performance of CNT-PA nanocomposite membranes is closely related to the uniform distribution of CNTs on the membrane surface—uniform distribution makes the membrane surface smoother, thereby reducing sites available for bacterial attachment [60,70,72]. Dynamic filtration experiments show that microbial counts in the influent and effluent of CNT-PA membranes are 35% and 31% lower than those of commercial membranes, respectively, confirming their antifouling advantage in practical operation [70,72].

MoS_2_ nanosheet- and g-C_3_N_4_ nanosheet-modified membranes similarly utilize the sharp edge structures exposed on the membrane surface to disrupt bacterial cell membranes via a similar physical piercing mechanism (as shown in Figure 5d) [79,84,85]. Among them, the atomic-scale sharpness of MoS_2_ edges endows it with stronger physical cutting capability, while g-C_3_N_4_ often combines physical puncture with photocatalytic ROS generation, achieving dual bactericidal effects [79,84,85].

#### 4.1.4. Anti-Adhesion Mechanism

Unlike other mechanisms that directly kill bacteria, the anti-adhesion mechanism is essentially a passive defense [5,10,15]. Even with efficient bactericidal ability, accumulation of dead bacterial debris on the membrane surface can still form a fouling layer and reduce flux. Therefore, constructing a barrier that prevents initial bacterial attachment is as important as bactericidal mechanisms.

**Figure 6:**
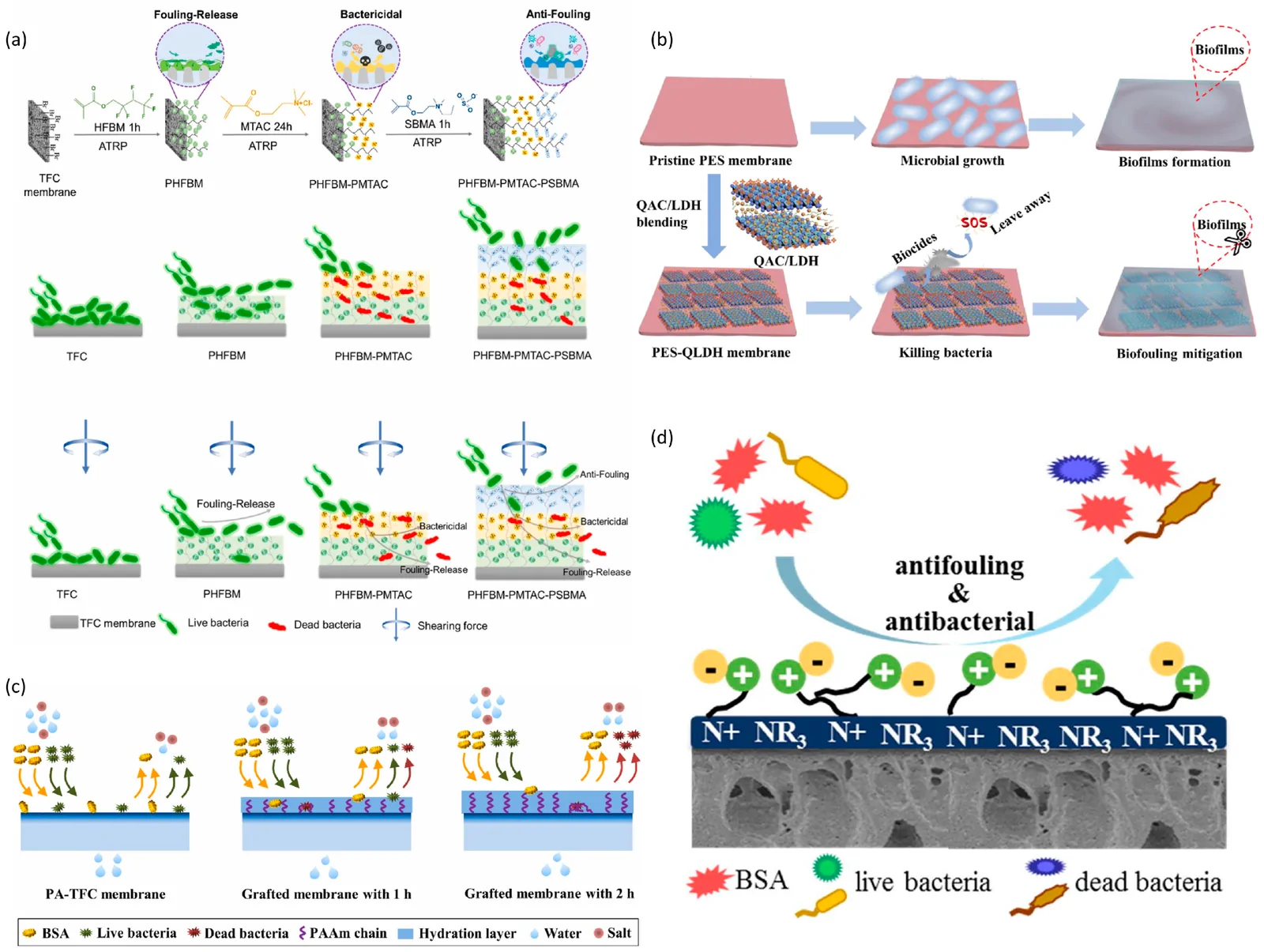
Schematic diagram of anti-adhesion and antibacterial mechanisms. (**a**) The preparation process of TFC-modified membrane and the different bacterial adhesion effects of different membrane formations [12]. (**b**) Preparation of guanidine polymer-modified membrane and schematic diagram of anti-adhesion [17]. (**c**) Schematic diagram of anti-adhesion effect of polyacrylamide-grafted membrane at different times [104]. (**d**) Schematic diagram of anti-adhesion and antibacterial mechanism of quaternary ammonium salt and zwitterionic polymer membrane [15].

TFC membrane modification involves sequentially grafting three functional polymers through atom transfer radical polymerization (ATRP) [12]. The preparation process consists of five steps (as shown in Figure 6a). In the static release experiment, the membrane surface was incubated with an *E. coli* suspension for 3 h. The attached bacteria were then detached by ultrasonication and quantified by plate counting. The membrane in Figure 6a released 73.0%, 84.4%, and 95.8% of adherent bacteria, respectively, indicating that the fluorinated low surface energy polymer endowed the membrane with excellent self-cleaning performance [12]. The pristine membrane exhibited a flux of 17.8 L·m^−2^·h^−1^·bar^−1^, while the modified membrane achieved 20.9 L·m^−2^·h^−1^·bar^−1^. In a 6 h dynamic biofouling experiment, the FRR improved from 79.1% for the pristine membrane to 96.5% for the modified membrane, indicating superior long-term antifouling stability [12].

The antibacterial process of guanidine polymer-modified membranes involves three aspects (as shown in Figure 6b) [17,73]. First, the guanidine groups grafted on the membrane surface firmly bind to the hydrophobic segments, thereby facilitating polymer insertion into the lipid bilayer. Simultaneously, they act on the cell wall through cation exchange, together disrupting both the cytoplasmic membrane and the osmotic balance. Second, guanidine polymers interfere with nucleic acid formation, inhibiting bacterial division and reproduction. Third, the polymer forms a film on the microbial surface that blocks respiratory channels [17,73].

Polymer-brush-modified membranes regulate the hydrophilicity/hydrophobicity and charge distribution of the membrane surface by adjusting the chain length, density, and charge characteristics of grafted chains (as shown in Figure 6c) [104,111]. Dense polymer brushes can create a steric hindrance barrier that prevents bacteria from approaching the membrane surface [35,104]. By interacting with the negative charges and hydrophobic components on the bacterial outer membrane, certain polymer brushes resembling natural antimicrobial peptides can cause disruption of the membrane structure [94,104].

Zwitterionic polymers bind water strongly via electrostatic interactions, forming a hydration layer that resists protein and bacterial adhesion [12,15]. Researchers combined zwitterionic polymers, quaternary ammonium salts, and PVDF into a modified membrane with synergistic antibacterial effects. The pure water flux reached 216 L·m^−2^·h^−1^·bar^−1^ [15]. FRR increased from 58.4% to 84.7%. Pore size decreased from 4.96 to 4.79 nm. Pore blocking was lowest for the zwitterionic membrane. In fluorescent protein adsorption tests, the quaternary ammonium-only membrane showed intense fluorescence due to high protein adsorption, while the zwitterionic-grafted surface appeared nearly dark, demonstrating excellent anti-adhesion (as shown in Figure 6d) [15].

### 4.2. Synergistic Effects Between Nanomaterials and the Membrane

The effective functioning of the above four antibacterial mechanisms relies on the synergy between nanomaterials and the membrane substrate, rather than operating in isolation (as shown in Table 3) [33,34,46]. The role of the membrane in the cooperative system mainly includes the following four aspects.

(1) The membrane provides a fixed platform for nanomaterials. Through coating, grafting, or blending, nanomaterials are anchored on the membrane surface or within the matrix, preventing loss and aggregation. The three-dimensional network structure of the membrane can limit the migration of nanomaterials, keeping antibacterial sites persistently exposed at the solid–liquid interface [5,10].

(2) The membrane regulates the release behavior of nanomaterials. In ion-release systems, the degree of encapsulation of nanomaterials by the membrane matrix determines the ion release rate. Core–shell structures (e.g., Ag@RF) and carrier loading (e.g., GO-Ag) both utilize the physical barrier effect of the membrane to achieve sustained release, controlling the release concentration within a safe threshold while maintaining antibacterial activity [40,49]. For example, the Cu leaching rate of thin-film composite (TFC) L-glutamine/Cu_3_-modified membranes was ≤3%, indicating significant immobilization of copper nanoparticles by the membrane matrix [106,112].

(3) Membrane structure affects the antibacterial efficiency of nanomaterials. When nanomaterials are more enriched on the membrane surface than in the interior, their contact probability with bacteria is higher, leading to higher antibacterial efficiency. Conversely, nanomaterials completely embedded in the dense active layer may not exert their full activity due to mass transfer limitations [29,31]. This synergy also explains why surface grafting or coating generally exhibits higher immediate antibacterial efficiency than matrix blending, although the latter may offer longer-term latent activity [29,31].

(4) The membrane and nanomaterials together determine the anti-biofouling performance. The hydrophilicity, charge, and roughness of the membrane surface affect the initial adhesion probability of bacteria, while the bactericidal activity of nanomaterials acts on the already attached bacteria [5,10]. Their synergy constitutes a complete defense line from anti-adhesion to killing, inhibiting membrane fouling at the source [5,10].

**Table 3:** Classification of main antibacterial mechanisms of nanomaterial-modified membranes.

Mechanism Type	Representative Materials	Mode of Action	Time Scale	Key References
Ion release	AgNPs, CuNPs	Metal ion release → enzyme inactivation/DNA damage	Hours to weeks, tunable by shell	[39,50,64,97]
ROS oxidation	TiO_2_, ZnO	Photocatalytic/chemical catalysis → ROS → cell oxidation	Seconds to minutes, requires light trigger for photocatalysis	[24,60,63,64]
Physical contact disruption	GO, CNTs, MoS_2_	Sharp edge cutting/piercing → content leakage	Instant, contact and activated	[69,76,110]
Anti-adhesion	Zwitterionic polymers, hydrophilic coatings	Lower surface energy/hydration layer barrier → prevent initial attachment	Continuous, passive defense	[12,14,104,113]

### 4.3. Target Bacterial Strains for Nanomaterial-Modified Membranes

Antibacterial performance evaluation of nanomaterial-modified membranes is typically conducted against common model bacterial strains in water treatment, covering both *Gram-negative* and *Gram-positive bacteria* [10,27,56], as shown in Table 4.

## 5. Conclusions and Perspectives

### 5.1. Conclusions

Membrane separation technology has been widely applied in water treatment due to its high efficiency and low energy consumption [117]. However, during long-term operation, microbial attachment, growth, and secretion of extracellular polymeric substances on the membrane surface gradually form a dense biofilm. This leads to decreased flux, reduced selectivity, and increased operating costs, representing a major bottleneck restricting the engineering application of membrane systems [118,119,120]. To address this challenge, researchers introduced antibacterial nanomaterials into the membrane structure to endow the membrane with active sterilization capability.

The antibacterial performance is largely determined by the dimensional characteristics of the nanomaterials. Zero-dimensional materials, such as Ag, Cu, and metal oxides, mainly rely on ion release and ROS generation for chemical disinfection, though they are prone to agglomeration. One-dimensional materials, like CNTs, achieve physical piercing via their high aspect ratio. Two-dimensional materials, such as GO and MXene, combine edge physical cutting with water channels between the layers. Three-dimensional materials, like MOFs, offer porous scaffolds for sustained release. Therefore, structural parameters of the materials, including size, edge exposure, and pore topology, are critical determinants of antibacterial activity and separation performance.

Regarding modification strategies, a clear trade-off exists between antibacterial efficiency and long-term stability. Surface coating maximizes exposure of antibacterial sites but is prone to detachment. Surface grafting achieves stable anchoring through covalent bonds but involves complex procedures. Matrix blending is easy to scale up but faces challenges in compatibility and leaching.

Finally, the synergistic interactions between nanomaterials and the membrane matrix establish a multi-mechanism antibacterial system. The four mechanisms not only function independently under membrane immobilization, release regulation, and structural guidance, but also work together to form a complete defense cascade from passive antifouling to active bacterial inactivation.

In summary, nanomaterial-modified antimicrobial membranes are not a simple additive composite, but a complex system where dimensional structure, modification strategies, and antibacterial mechanisms are tightly coupled. The choice of modification strategy directly affects antibacterial efficiency and long-term stability, while the synergy of multiple mechanisms provides theoretical support for the rational design of high-performance antimicrobial membranes.

### 5.2. Challenges and Countermeasures

Current antibacterial membrane research still faces three major challenges [4,5,114].

First, the insufficient interfacial compatibility between inorganic nanoparticles and organic polymer matrices remains a critical issue that needs to be solved. High-surface-energy nanomaterials tend to agglomerate in the casting solution, forming non-selective defects that reduce separation accuracy and mechanical stability [114]. Strategies such as dopamine coating or silane coupling agent modification can introduce compatible functional groups, effectively inhibit agglomeration and enhance interfacial bonding, thus laying a foundation for preparing high-performance composite membranes.

Secondly, some modified membranes may experience a decline in flux, rejection rate, pore blocking, and gradual loss of antibacterial activity after long-term operation. To address these problems, future research can optimize membrane structure design, carry out periodic chemical cleaning, or develop online repair technologies to slow down performance degradation [121,122,123].

Finally, the lack of a standardized evaluation system for industrial applications has seriously hindered the translation of this technology into practice. Most current studies only report short-term static antibacterial rates, without systematic assessments of flux recovery, cumulative ion release, or membrane module lifespan under dynamic operating conditions [19,24]. To solve this, the evaluation system should extend cross-flow operation cycles, include multiple fouling–cleaning cycles, and track the mass balance of nanomaterials, post-aging performance, and ecotoxicity. In large-scale production, comprehensive consideration must be given to the cost-effectiveness of nanomaterials, the reproducibility of the preparation process, strategies for maintaining long-term stability of membrane modules, and environmental risks throughout the entire life cycle. The synergistic integration of all the above strategies will be a key pathway to promote antibacterial membrane technology from laboratory research to practical engineering applications.

### 5.3. Perspectives

The future development of antibacterial membranes will show a convergence of material innovation and system integration. The performance optimization of single nanomaterials is approaching its limit, and multi-dimensional synergistic design will become a breakthrough direction [29,31]. For example, 2D GO nanosheets on the membrane surface provide an immediate physical barrier and fast water channels, while 0D AgNPs in the matrix provide long-term ion release. The two complement each other on different timescales. The 2D sheets enriched on the surface are responsible for physical sterilization and molecular sieving, while the 0D particles dispersed inside provide sustained release. This synergistic approach may overcome the performance ceiling of single materials [32,33,118].

On this basis, the development of intelligent responsive antibacterial films is becoming a new trend. By encapsulating antibacterial agents in pH, temperature, and light-sensitive carriers, a release mode can be triggered only when bacterial infection or biological contamination occurs, which not only prolongs membrane life but also reduces environmental risks. At the same time, the introduction of high-throughput computing screening and intelligent membrane design can accelerate the discovery of new nanomaterials or polymer combinations, efficiently predict the correlation between membrane structure and performance, significantly shorten the traditional trial-and-error cycle, and combine automated membrane preparation and online characterization technology, which is expected to achieve rapid iteration and precise optimization of antibacterial membrane formulations. Although water treatment remains the main application scenario for antibacterial membranes, their design concepts are increasingly expanding into fields such as surface modification of biomedical implants, wound dressings, and active food packaging. These emerging scenarios require higher biocompatibility and antibacterial persistence, which will promote the development of low-toxicity nanomaterials and degradable polymer matrices. In addition, environmentally friendly design and low-carbon manufacturing processes throughout the entire lifecycle will also become inevitable choices in the industrialization process.

The mutual empowerment and collaborative promotion of material innovation, intelligent manufacturing, and application expansion will jointly constitute the core research content of the future antibacterial membrane field, promoting the leap from laboratory achievements to practical engineering applications.

## Figures and Tables

**Table 1:** Antibacterial effects of nanomaterials of different dimensions.

Dimension	Representative Materials	Key Structural Variables	In-Membrane State/Position	Antimicrobial Contribution	Main Limitations	Key References
0D	AgNPs, ZnO,	Particle size, surface coating, defects	surface enrichment or matrix embedding	ion release, contact sterilization	agglomeration, burst release, excessive leaching, and deactivation of active sites due to polymer embedding	[39,40,48,64,99,100]
1D	CNTs	Diameter, length, aspect ratio, orientation, surface functional groups	surface exposure, directional alignment, entanglement and agglomeration, or polymer encapsulation	physical puncture of cell membrane, structural defect induction, carrier function	entanglement and agglomeration, difficult orientation control, polymer coating on the surface, insufficient exposure	[28,68,69,72]
2D	MXene, GO	Horizontal dimensions, number of layers, edge density, interlayer spacing, surface terminal groups	surface coating, layer-by-layer stacking or matrix embedding	edge-mediated physical puncture, photocatalysis, electrostatic anti-adhesion	re-stacking, edges encapsulated by polymers, interlayer spacing swelling or collapse, increased transmission resistance	[1,46,48,49,57,77,89,90]
3D	MOFs	Pore size/pore topology, metal node type, particle size, defects, framework stability	surface immobilization or matrix embedding	metal node hydrolysis/ligand exchange for slow-release bactericidal ions, ligand-assisted antibacterial activity	metal/ligand leaching, poor interfacial compatibility with polymers, mass transfer resistance	[28,91,95,96,98]

**Table 2:** Comparison of immobilization strategies for antibacterial nanomaterials in membranes.

Immobilization Strategy	Combination Method	Main Advantages	Main Limitations	Long-Term Operational Stability	Key References
Chemical grafting	Covalent bonding of nanoparticles onto membrane	Firm attachment, precise positioning, no membrane damage	Complex steps, risk of damaging selective layer	High (covalent bonds prevent leaching)	[94,98,104,106]
Polymer coating	Physical/weak chemical encapsulation of nanoparticles in polymer layer	Protects Nanoparticles, controls release, improves dispersibility	Coating may rupture; may reduce antibacterial activity	Moderate (depends on coating integrity)	[51,54,76]
Core–shell structure	Active core (e.g., Ag) with slow-release shell (e.g., RF resin)	Sustained release, anti-leaching, long-term efficacy	Shell may hinder microbe contact; complex synthesis; thick shell reduces permeability	High (shell acts as barrier, but shell stability needs evaluation)	[35,66,75]
Surface functionalization	Ligand modification of NP surface for better dispersion and adhesion	Improves dispersion, reduces aggregation	Functional layer may dissolve in certain environments; leaching risk	Medium (depends on binding strength)	[16,18,45]
Carrier blending	Direct mixing of NPs with casting solution	Simple, scalable, no complex chemistry	Nanoparticles aggregate, weak binding, pore blockage, easy leaching	Poor (physical fixation, highest leaching risk)	[9,62,91,96]

**Table 4:** Main target bacterial strains and representative references.

Microbial Model	Main Membrane Types	Nanomaterial Loading	Test Conditions	Antibacterial Endpoint	Actual Relevance	Key References
*Escherichia coli*	Ag@RF nanocomposite nanofiltration membrane	0.02 wt% Ag@RF	Viable plate count	Mainly viable cell reduction	*Gram-negative* and a common indicator bacterium in water bodies.	[30,40,49,52,56,57,106]
*Staphylococcus aureus*	Ag-MOFs PES membrane	0.1 wt% Ag-MOFs	Live/Dead cell staining	Mainly viable cell reduction and growth inhibition	*Gram-positive* pathogenic bacteria, model organisms for skin and hospital infections.	[47,49,57,104,105,106]
*Bacillus subtilis*	rGO/ZnO-z-PES membrane	5 wt%rGO/ZnO	Static/dynamic adhesion assay	Mainly adhesion reduction	*Gram-positive*, spore producing, highly resistant environmental biofilm model.	[17,73,104]
*Pseudomonas aeruginosa*	rGO/ZnO-z-PES membrane	5 wt%rGO/ZnO	Continuous-flow biofilm reactor	Mainly biofilm reduction	*Gram-negative* opportunistic pathogens, robust biofilm forming agents, and models of chronic biofilm infections.	[27,113]
*Pseudomonas fluorescens*	RO	–	RO cross flow reactor	Mainly biofilm reduction	Common environmental biofilm forming bacteria isolated from self-contaminated membrane.	[114]
*Sphingomonas-dominated community*	RO	–	Operating in a full-scale RO water plant	Mainly biofilm reduction	The key EPS producing pioneer colonizer that initiates and dominates RO membrane biofilm under oligotrophic conditions.	[1,32,115]
Industrial RO membrane community	RO	–	Lab-scale RO system, constant flux of 30 LMH, continuous TMP monitoring	Viable cell reduction and biofilm reduction	The mixed species community of the full-scale water plant represents the actual pollution scenario.	[116]

## Data Availability

No new data were created or analyzed in this study. Data sharing is not applicable to this article.
